# Case Report: Nephrocalcinosis from pancreatic hypoplasia in HNF1B disease: a multigenerational expression with genetic confirmation in the youngest generation

**DOI:** 10.3389/fmed.2025.1671893

**Published:** 2026-01-30

**Authors:** Naga Sumanth Reddy Gopireddy, Sahil Grover, Imran Khawaja, Mohamed Fawzi Mudarres, Prerna Rastogi, Maria Story, Christie P. Thomas

**Affiliations:** 1 Division of Nephrology, Department of Internal Medicine, University of Iowa Hospitals and Clinics, Iowa city, IA, United States; 2 Department of Pathology, University of Iowa Hospitals and Clinics, Iowa City, IA, United States; 3 Division of Nephrology, Department of Internal Medicine, Southeast Iowa Regional Medical Center, West Burlington, IA, United States

**Keywords:** HNF1B mutation, renal cysts and diabetes syndrome (RCAD), maturity-onset diabetes of the young (MODY), pancreatic hypoplasia, nephrocalcinosis, hyperoxaluria, chronic kidney disease (CKD), genetic kidney disease

## Abstract

**Background:**

HNF1B-associated disease is a genetic disorder caused by heterozygous pathogenic variants in the HNF1B gene, leading to a variety of clinical phenotypes primarily affecting the kidneys, pancreas, liver, and genitourinary tract. First identified in 1997 as the cause of MODY5 (maturity-onset diabetes of the young, type 5), the disease spectrum has since expanded to include kidney disease, pancreatic hypoplasia, genital malformations, gout, hypomagnesemia, liver abnormalities, and primary hyperparathyroidism. Variable expressivity and limited penetrance complicate diagnosis, with some individuals presenting without the classical features of partial pancreatic hypoplasia, renal developmental abnormalities or MODY. Genetic testing remains crucial for accurate diagnosis, especially in families with varied phenotypic expressions.

**Case Presentation:**

The index case, a 53-year-old woman, presented with hyperoxaluria, advanced chronic kidney disease (CKD), bilateral nephrocalcinosis, and pancreatic hypoplasia. Family members demonstrated diverse features, including renal cysts, kidney stones, diabetes, and pancreatic dysfunction. Genetic testing confirmed a pathogenic HNF1B variant (C295R) in the youngest family member, reinforcing the hereditary nature of the condition.

**Conclusion:**

This case report highlights the variable clinical presentation of HNF1B nephropathy across generations, illustrating the multisystem nature of the disease and emphasizing the importance of genetic testing for diagnosis and family screening.

## Introduction

The hepatocyte nuclear factor 1 beta (HNF1B) protein, encoded by the *HNF1B* gene, is a transcription factor that plays a crucial role in the development and functioning of various organs, particularly the kidney and pancreas. The *HNF1B* gene is expressed in the epithelium of several tissues, including the kidney, pancreas, liver, and genitourinary tract. It is also transiently expressed in the neural tube, epididymis, seminal vesicles, prostate, and uterus. Loss-of-function variants in *HNF1B* can lead to multiple phenotypes (1).

*HNF1B* was initially identified in 1997 as the gene responsible for one form of maturity-onset diabetes of the young (MODY5) (1). Subsequent clinical observations in families affected by MODY5 showed that *HNF1B* gene variants were also associated with cystic renal disease, and the genetic syndrome was initially described as Renal Cysts and Diabetes (RCAD) (2, 3). However, patients may also present with involvement of the kidneys, pancreas, liver, electrolyte imbalances, and genital malformations—such as vaginal aplasia, bicornuate uterus, and atresia of the vas deferens—without necessarily exhibiting renal cysts or diabetes (4).

**Figure 1:**
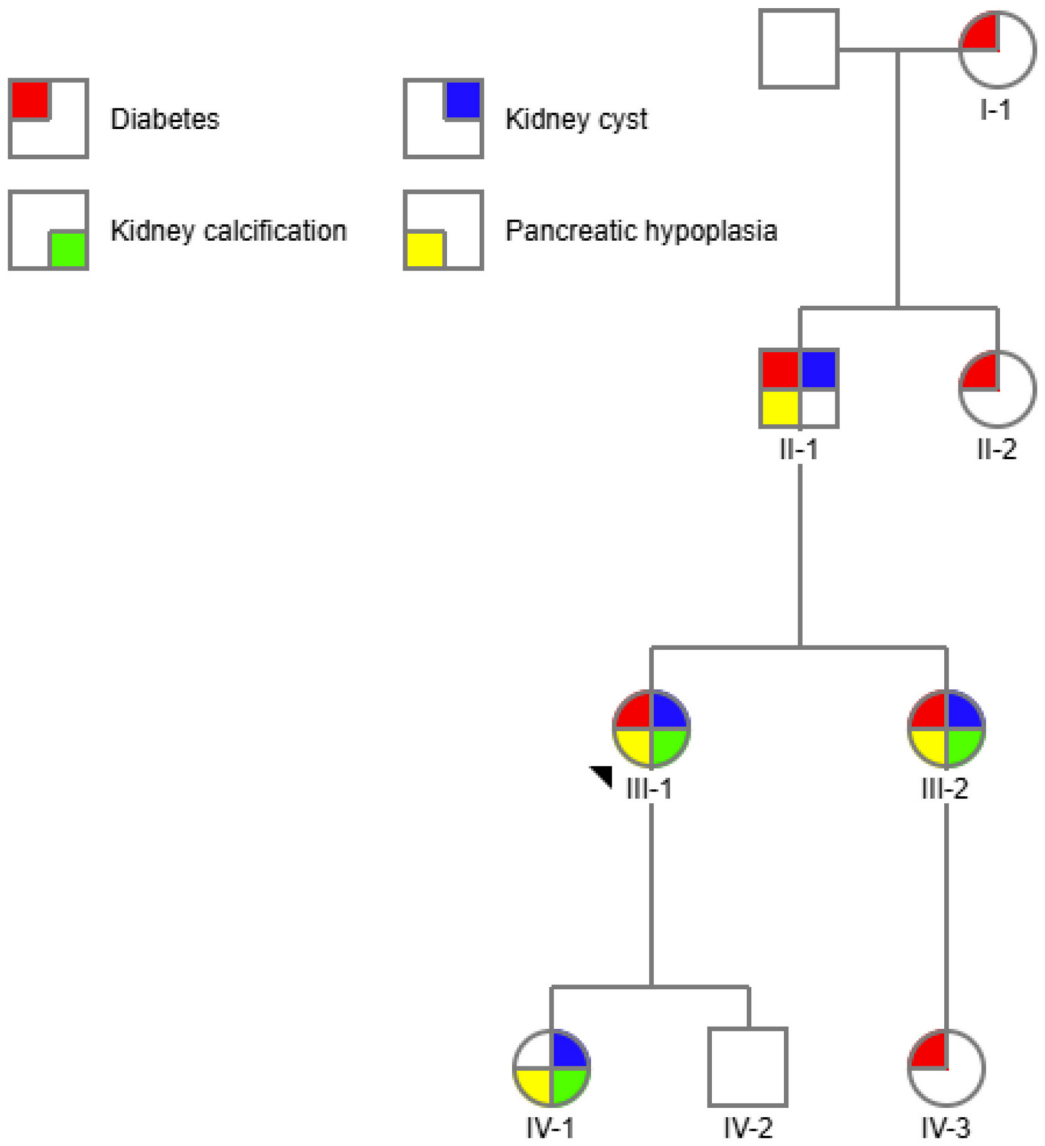
Pedigree chart. 4 generation family shown with quadrants or sectors to demonstrate 4 phenotypes in affected individuals. The proband is III-1.

*HNF1B*-associated disease is caused by heterozygous variants and is therefore characterized by autosomal dominant inheritance. However, up to 50% of cases arise from *de novo* genetic variants. Furthermore, expressivity can be variable, with some family members manifesting diabetes or exocrine pancreatic disease and others presenting with kidney phenotypes. We present multiple family members within a single family across generations (Figure 1), each with different phenotypic characteristics, highlighting the diagnostic dilemmas posed by HNF1B-associated disease even when multiple family members are involved.

### Individual 1 (Proband)

**Figure 2:**
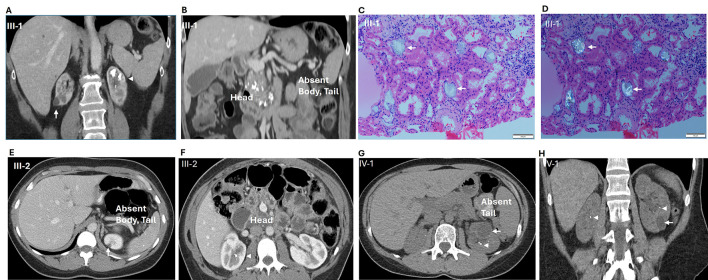
**II-1**: **A**- Kidney cysts and Nephrocalcinosis, **B** - Calcifications in head of pancreas, absent pancreas body and tail, **C** (renal biopsy) - H & E-stained section, Magnification 20X. Sections shows tubules with non-basophilic microcalculi (arrows), **D** (renal biopsy) - Section viewed under a polarizer shows bright, polarized oxalate microcalculi (arrows); **III-2**: **E**- Absent pancreas body and tail; **III-2**: **F** - Cysts and calculi in the head of pancreas and renal calculi ; **IV-1**: **G**- Absent pancreatic tail and renal cysts and calculi; **IV-1**: **H** - Bilateral renal cysts and calculi. Arrows point to kidney cysts; Arrowheads point to kidney calcifications in panels A, E, F, G and H.

A 53-year-old woman with a long-standing history of diabetes mellitus (previously attributed to Type 2 DM) for at least 25 years, presented in April 2012 with progressive kidney dysfunction. Despite excellent glycemic control on insulin, her kidney function deteriorated significantly over several months. A kidney biopsy revealed mesangial expansion, consistent with diabetic nephropathy, alongside intratubular calcium oxalate depositions, which raised suspicion for hyperoxaluria (Figures 2C, D, III-1). The patients' family history was remarkable for kidney disease affecting her father and sister, suggesting an underlying genetic kidney disease. Three cousins had been diagnosed with cystic fibrosis.

She was diagnosed with CKD stage 5 with nephrocalcinosis, hyperparathyroidism, metabolic acidosis and anemia due to CKD, attributed to underlying diabetes with possible contribution by hyperoxaluria. Additional workup, including 24-h urinary oxalate studies, showed high urine oxalate (178 mg/dl, normal range <40 mg/dl) and low urine citrate (<28 mg/dl) while genetic testing for AGXT variants, associated with primary hyperoxaluria type 1 (PH1), returned negative. CT abdomen and pelvis revealed calcifications in the pancreatic head, atrophy or agenesis of the pancreatic body and tail, and multiple kidney stones and kidney cysts (Figures 2A, B, III-1). The persistence of high urinary oxalate level and pancreatic calcifications and partial agenesis on imaging suggested secondary hyperoxaluria due to malabsorption from pancreatic insufficiency.

In December 2012, the patient underwent a living-unrelated kidney transplant. Over the next decade, her transplant function remained stable but was complicated by hypertension, proteinuria, and anemia. Pyridoxine, a low-oxalate diet, and erythropoietin were used to manage hyperoxaluria and anemia. By February 2022, her creatinine had risen to 2.5 mg/dl.

10 years later, the patient developed progressive neurological complications and work up led to a diagnosis of EBV-associated lymphoproliferative disorder in the brain. In February 2024, her condition worsened with respiratory failure and cardiac arrest, revived, and she was transitioned to comfort care and discharged home with hospice support.

The patient's clinical journey highlights the complex and multi-systemic manifestations of HNF1B disease, which include progressive CKD with multiple kidney cysts and nephrolithiasis, diabetes due to partial agenesis of the pancreatic body and tail, and calcifications of the pancreatic head. Her case serves as the cornerstone of this family's phenotypical spectrum of HNF1B-related disease. Further exploration of her relatives' presentations will contextualize the inheritance and variability of this condition.

### Proband's father

A 70-year-old man with a long-standing history of diabetes mellitus presented in October 2005 with progressive jaundice, pale stools, dark urine, and intense pruritus, primarily in the evenings. He was identified with a cholestatic pattern on liver function tests and a hepatic hilar mass on ultrasound. He was referred to an outside hospital for further evaluation and management.

Advanced imaging with MRI of the abdomen and MRCP revealed a 3 cm infiltrative tumor involving the hepatic hilum. Biopsy via ERCP confirmed adenocarcinoma. The tumor was deemed unresectable. And the patient was advised to undergo external beam radiation therapy in combination with 5-fluorouracil. A biliary stent was placed.

The patient's past medical history was significant for diabetes mellitus, diagnosed 40 years earlier and managed with insulin, as well as hypertension. Imaging studies performed during his diagnostic workup revealed incidental findings of bilateral benign kidney cysts, some hemorrhagic, with bilateral kidney atrophy and a hypoplastic pancreatic tail.

Proband's father presentation highlights several phenotypic features consistent with an HNF1B disease. His kidney abnormalities, including bilateral cysts, align with the well-recognized renal phenotype of HNF1B mutations. His long-standing diabetes mellitus may represent maturity-onset diabetes of the young (MODY5), a common endocrine manifestation of HNF1B disease.

### Proband's sister

A 41-year-old woman came in initially with right upper quadrant discomfort radiating to her back. She also reported frequent, watery diarrhea. Her medical history was notable for diabetes, diagnosed at age 4 and managed with insulin since then.

Her initial lab results showed mild liver enzyme elevations (AST 65 IU/ml, alkaline phosphatase 170IU/ml) with normal kidney function, electrolytes, and pancreatic enzymes. CT abdomen showed a possible mass in the pancreatic head, along with calcifications in the pancreatic head and hypoplasia of the body and tail and multiple kidney stones (Figures 2E, F, III-2), Follow-up MRI abdomen and MRCP showed that she was missing the body and tail of her pancreas. Pancreatic enzyme replacement was started to help with exocrine insufficiency.

The constellation of findings in this patient, including partial pancreatic agenesis, early-onset diabetes, kidney cysts, nephrolithiasis, and chronic calcific pancreatitis, raises the possibility of an underlying HNF1B (hepatocyte nuclear factor 1-beta) variant as a unifying diagnosis.

### Proband's daughter

Proband's 32-year-old daughter presented to the renal genetics' clinic after the discovery of reduced kidney function and bilateral kidney cysts during her pregnancy. Laboratory findings at that time revealed mild serum creatinine elevation (1.5–1.6 mg/dl), elevated blood pressure, and trace proteinuria.

Following delivery renal ultrasound revealed bilateral kidney cysts, intrarenal calcifications, and mild asymmetry in renal size (right kidney: 9 cm; left kidney: 11 cm) (Figures 2G, H, IV-1). In the context of the family history this raised the suspicion of an inherited renal condition. Genetic testing identified a heterozygous pathogenic HNF1B variant (cysteine-to-arginine substitution at position 295), confirming the diagnosis ofHNF1B nephropathy or Renal Cysts and Diabetes (RCAD) syndrome. This diagnosis explained her clinical presentation, including kidney cysts, chronic kidney dysfunction, and mildly elevated liver enzymes (alkaline phosphatase and GGT), which were consistent with mild cholestasis. CT abdomen shows hypoplastic pancreatic tail and bilateral renal cysts with nephrolithiasis.

The patient's diagnosis highlights the systemic impact of HNF1B nephropathy, with kidney, metabolic, and potential pancreatic and hepatic manifestations, as well as its familial implications. The identification of the HNF1B variant offers a genetic explanation not only for her symptoms but also for the kidney disease and metabolic abnormalities observed in her maternal relatives, further reinforcing the importance of family history in identifying hereditary conditions.

**Figure 3:**
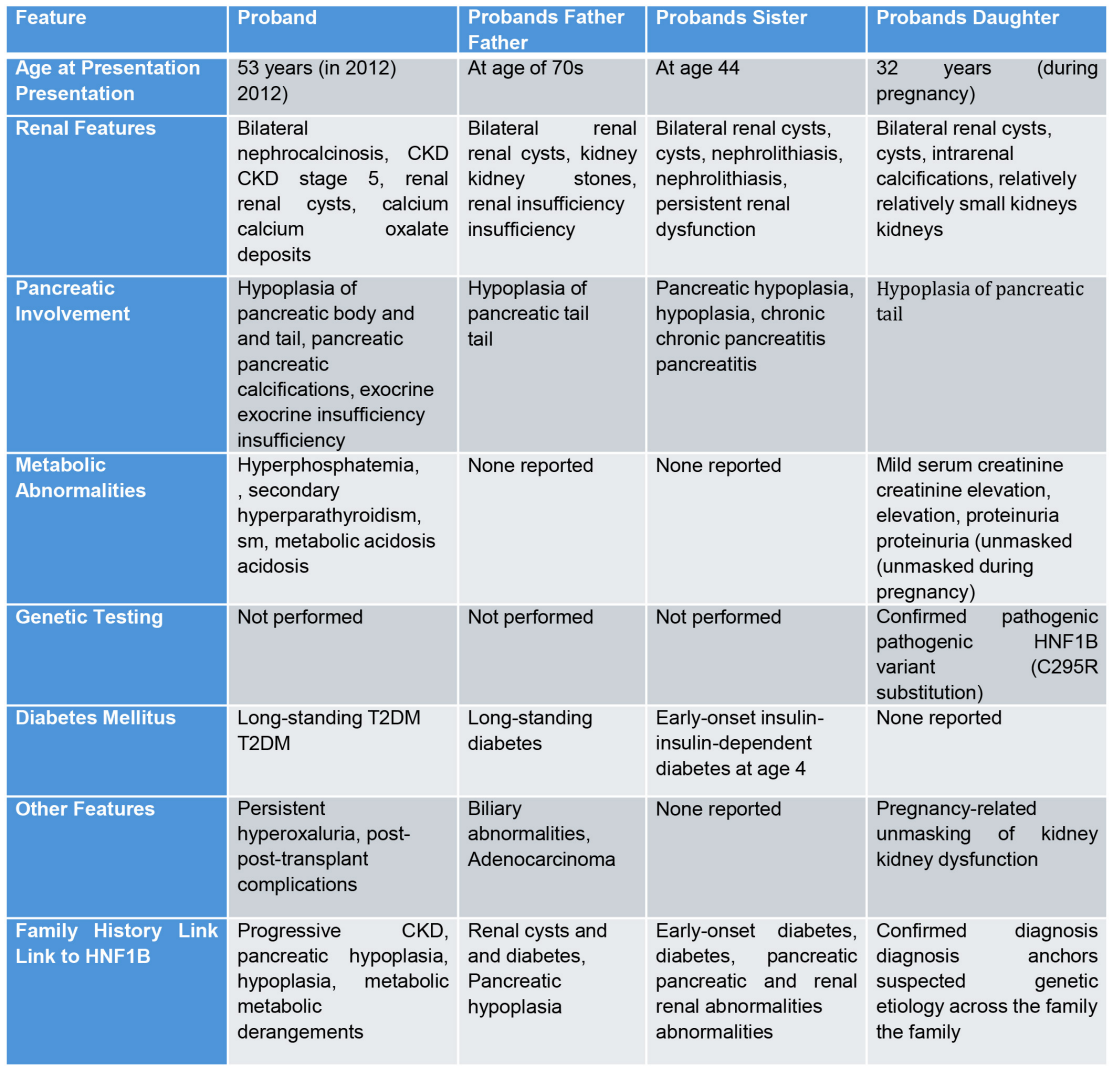
HNF1B case comparison.

Note: As these cases represent a retrospective compilation of clinical data collected over several years and across multiple encounters, consistent documentation of contemporaneous physical examinations was not available for all family members. Furthermore, no distinctive or clinically significant physical findings directly attributable to HNF1B-associated disease were noted during their evaluations. Consequently, detailed physical examination findings have not been included in this report, as they were not contributory to the diagnostic assessment or longitudinal interpretation of each case (Figure 3).

### Timeline of presentation of cases

The proband presented with diabetes in her mid-20s, initially resembling MODY, and renal biopsy revealed diabetic changes with nephrocalcinosis. Although the diagnosis of HNF1B-associated disease (MODY-5) was not established until her 50s, retrospective it became apparent that her presentation in her 20s was consistent with the phenotype. Her father developed diabetes around age 30, and MODY-5 features were only recognized retrospectively in his late 70s following the proband's diagnosis. The proband's sister was diagnosed with diabetes at age 4, initially presumed to have type 1 diabetes; however, she later had kidney stones, renal cysts, and was found to have a hypoplastic pancreas in her 40s. The proband's daughter was incidentally discovered to have multiple renal cysts at age 32 during pregnancy, along with a hypoplastic pancreatic tail and nephrolithiasis, though she remains euglycemic. This familial pattern illustrates the inconstant age of onset, the variable expressivity and the diverse phenotypic spectrum characteristic of HNF1B-related disease.

## Discussion

The first case report of *HNF1B* variant was described in Japanese family with MODY in1997. HNF1B-associated disease is most often inherited in an autosomal dominant manner, although up to 50% of cases arise from *de novo* mutations, thus frequently occurring without an apparent family history (4).

**Figure 4:**
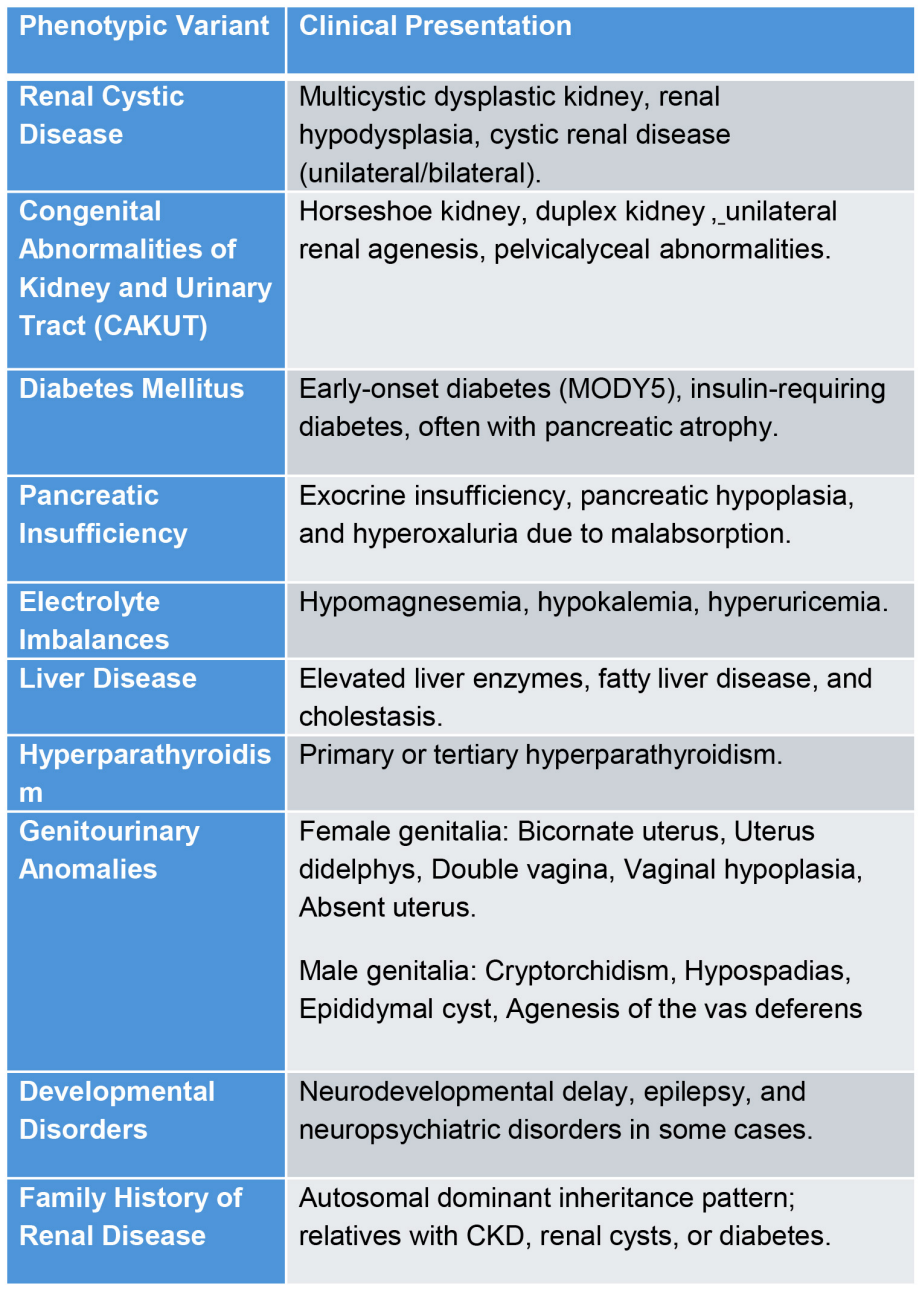
Phenotypic variants and presentations of HNF1B variants.

Approximately half of all cases involve a complete gene deletion, often as part of the 17q12 micro deletion syndrome, which affects14 additional genes. Neurological symptoms seen in some patients are likely attributable to this microdeletion rather than *HNF1B* alone. Both whole-gene deletions and truncating variants result incomparable phenotypes, suggesting haploinsufficiency as the primary pathogenic mechanism (5, 20). HNF1B variants can involve multiple organs and cause metabolic imbalances (Figure 4).

### Kidney presentation in *HNF1B* variants

Renal involvement is the most common finding in *HNF1B* disease with varied phenotypic presentations ranging from developmental renal disease to polycystic kidney disease to tubulointerstitial kidney disease and sometimes isolated hypomagnesemia. HNF1B nephropathy predominantly presents as a chronic tubulointerstitial disease featuring bland urinalysis, absence of hematuria and low-grade tubular proteinuria. Renal biopsy can show interstitial fibrosis, enlarged glomeruli or oligomeganephronia and enlarged tubules, implying impaired tubular energy balance (6). CKD progression is usually slow (approximately 2.45 ml/min/year), with rapid progression to ESRD occurring in around 20 % of cases.

Congenital anomalies of kidney and urinary tract (CAKUT) are common, where usual manifestations are vesicoureteral reflux, ureteropelvic junction abnormalities, multi cystic dysplastic kidneys and unilateral renal agenesis. HNF1B may accounts for as much as 10% CAKUT (7). Although underdiagnosed, HNF1B variants are found in approximately 10% of CKD cases with unexplained etiology.

Other kidney diseases such as familial glomerulocystic kidney disease (GCKD) which is a dominantly inherited condition characterized by glomerular cysts and variable renal size and function and familial juvenile hyperuricemic Nephropathy (FJHN), where hyperuricemia was seen with dominantly inherited tubulointerstitial kidney disease are now recognized as part of clinical spectrum of renal manifestations of HNF1B deficiency disease (6).

Nephrolithiasis and nephrocalcinosis as seen in this pedigree are not a well described kidney manifestation of HNF1B disease. HNF1B mutations can cause pancreatic insufficiency, leading to fat malabsorption. This reduces calcium binding to oxalate in the gut, increasing oxalate absorption (enteric hyperoxaluria). Excess urinary oxalate combines with calcium, promoting calcium oxalate deposition and nephrocalcinosis.

### Diabetes and pancreatic manifestations in *HNF1B* variants

Variants in genes for transcription factors HNF-1α and HNF-4α are linked to form of diabetes called MODY. This type of diabetes is inherited in an autosomal-dominant pattern, often appearing before age 25, and is characterized by problems with insulin secretion in response to glucose (1). Mutations in the *HNF1B* gene (initially identified while studying monogenic diabetes) are now recognized as a cause of both diabetes and kidney developmental disorders (8).

Diabetes in HNF1B mutation carriers varies widely, from relative euglycemia managed by diet to insulin-dependent diabetes. High blood sugar typically develops in adolescence or early adulthood due to reduced insulin secretion and increased resistance, resembling other MODY types. Varied long term outcomes were associated with HNF1B related diabetes, with one study concluded that 70% required insulin during follow up visits, although though no complications were noted and another study revealed that around 40% developed retinopathy or neuropathy after mean diabetic duration of 15 years (9). HNF1B-associated diabetes may present as new-onset diabetes after transplantation (NODAT), often triggered by weight gain and immunosuppressive therapy (10, 18).

Significant changes in pancreatic morphology have been identified mainly through imaging studies (CT, MRI, and MRCP), with limited histopathological data available. Imaging findings highlight the absence of the pancreatic body and tail, as well as the accessory and dorsal ducts, suggesting agenesis of the dorsal pancreas. The ventral bud and pancreatic head are generally preserved but may show partial involvement (11). Unlike the cystic dilatations observed in extrahepatic bile ducts, none were reported in the pancreas. Parenchymal calcifications were occasionally noted but showed no consistent correlation with clinical or morphological features, though they may be linked to late-phase dysfunction and diabetes development (12).

The findings from this study reveal significant variability in the clinical presentation of pancreatic and metabolic involvement within a single family spanning multiple generations. While two individuals exhibited pancreatic hypoplasia with exocrine dysfunction, three family members were diagnosed with diabetes mellitus, including one with an unusually early onset at the age of 4 years. Interestingly, not all family members shared these manifestations, as some did not develop diabetes despite their shared genetic background.

### Electrolyte abnormalities in HNF1B variants

The most frequent electrolyte abnormalities encountered in HNF1B variants are hypomagnesemia, hypokalemia and hyperuricemia (13). Nearly 50% of patients with HNF-1β mutations experience hypomagnesemia due to kidney magnesium (Mg^2+^) wasting (15). The primary sites for Mg^2+^ reabsorption in the kidney are the thick ascending limb (TAL) of the loop of Henle and the distal convoluted tubule (DCT) (14). Impaired Mg^2+^ handling in the TAL often results in the concurrent loss of calcium (Ca^2+^), while defects in the DCT are associated with hypermagnesuria is seen without concurrent urine calcium loss. In patients with HNF1B mutations, hypomagnesemia is usually accompanied by hypocalciuria (13). However, some adults exhibit normocalciuria, suggesting that Mg^2+^ reabsorption impairment is primarily localized to the DCT. Recent studies have shown that HNF-1β functions as a transcriptional activator of the calcium-sensing receptor (CaSR), which is critical for Ca^2+^ regulation in the TAL. Reduced CaSR activity, because of HNF1B haploinsufficiency, may contribute to the observed hypocalciuria in these patients.

Faguer et al. reported that 46% of adults with HNF1B mutations exhibited hypokalaemia (13). This condition is often linked to Mg^2+^ deficiency, as reduced intracellular Mg^2+^ levels can relieve the inhibition of the renal outer medullary potassium channel (ROMK), resulting in increased urinary potassium (K^+^) loss.

Proximal tubule (PT) dysfunction is infrequently observed in patients with HNF1B variants, but sometimes hyperuricemia is present and accompanied by reduced fractional excretion of uric acid (15, 19).

### Liver and HNF1B

The initial characterization of HNF1B deficiency as a syndrome primarily involving kidney disease and diabetes has led to the under recognition of liver involvement. As a result, liver involvement remains poorly studied and has largely been reported as either an asymptomatic elevation of transaminase levels or, less commonly, as cholestatic liver disease, especially neonatal cholestasis (16, 17).

The hepatic phenotype in HNF1B mutation positive patients' clusters into three diverse levels of severity, ranging from severe neonatal cholestasis through late-onset cholestasis in middle-aged adults up to a mild hepatic phenotype on the background of other, more pronounced symptoms and signs, such as diabetes and kidney disease. Interestingly, despite HNF1B deficiency predominantly affecting the biliary epithelium, only hepatocellular carcinoma (HCC) has been observed thus far, with no reported cases of adenocarcinoma (14).

In our series, two patients exhibited liver involvement. One patient presented with obstructive jaundice and was diagnosed with an adenocarcinoma. The second patient had asymptomatic elevations in transaminases and gamma-glutamyl transferase (GGT) without any other significant liver-related manifestations.

### Other systemic manifestations

Mutations in *HNF1B* are associated with diverse extrarenal manifestations, including reproductive abnormalities. In females, these abnormalities can range from incomplete fusion of the Müllerian ducts—manifesting as bicornuate uterus, uterus didelphys, or double vagina—to absent uterus and vaginal hypoplasia. Male genital abnormalities are infrequent but include cryptorchidism, hypospadias, epididymal cysts, and agenesis of the vas deferens (16).

Certain additional phenotypes are specifically linked to a 17q12 microdeletion encompassing *HNF1B*. While early studies focused solely on *HNF1B* deletions, subsequent research revealed that most cases with complete HNF1B deletions involve a common 17q12 microdeletion. This deletion is associated with distinct features beyond the manifestations of *HNF1B* haploinsufficiency alone, including developmental delay, intellectual disability, autism spectrum disorder, schizophrenia, anxiety, and bipolar disorder (16).

## Conclusion

This case series illustrates the broad phenotypic variability and diagnostic challenges of HNF1B-associated nephropathy, highlighting its relevance as a key consideration in patients with unexplained kidney dysfunction and multisystem involvement. The distinctive features of HNF1B mediated disease in this case report is the coexistence of pancreatic calcification and nephrocalcinosis highlighting a distinctive HNF1B phenotype linking enteric hyperoxaluria from pancreatic insufficiency with nephrocalcinosis.

While rare, HNF1B mutations are increasingly recognized as a cause of renal and extrarenal abnormalities, underscoring the importance of an integrated diagnostic approach including genetic testing. The observations in this multigenerational family can anchor a genetic diagnosis.

HNF1B mutations exhibit broad phenotypic diversity, including kidney cysts, CKD, nephrocalcinosis, and secondary hyperoxaluria, along with systemic features like pancreatic hypoplasia, diabetes, and metabolic abnormalities. A strong family history can reveal autosomal dominant inheritance, prompting timely genetic evaluation. In women, pregnancy may unmask underlying HNF1B-related kidney disease. Genetic testing—such as confirmation of the C295R variant—helps unify multisystem findings, supports diagnosis, and informs management and counseling. Given the syndrome's multi-organ involvement, multidisciplinary collaboration is crucial for optimal patient care.

Patient perspective: Two of the 4 people in this family described here have passed on. The youngest member of the family described here did not have any inkling that the family had a genetic disease even though some of them had advanced disease. A genetic disease had not yet been formally established at the time, nor had there been open discussion among family members about that possibility. When the proband's daughter was noted to have elevated kidney function during her pregnancy that did not resolve post-delivery and subsequent investigation revealed kidney cysts, she was referred to the renal genetics' clinic. It was heartbreaking for her to learn that she had a genetic condition called HNF1B nephropathy and that this genetic disease had affected her mother and other family members. She continues to be asymptomatic and is adjusting to her disease. She has decided to limit her children after the second of her children was identified with disease in utero. The identification of a genetic disease has provided clarity about her disease and helped her understand the possible course of disease and the possible risk to her children.

As an educational point, a clinician should suspect HNF1B-mediated disease in patients presenting with early-onset diabetes especially if there is a family history, or absence of the body and tail of the pancreas with or without pancreatic insufficiency. HNF1B mediated disease should also be considered when there is atypical polycystic kidney disease, autosomal dominant tubulointerstitial kidney disease, abnormalities in kidney and urinary tract development like unilateral renal agenesis or multicystic dysplastic kidneys or isolated hypomagnesemia. Suspicion for HNF1B mediated disease should be further strengthened by the present of both pancreatic and kidney disease with or without hepatobiliary or genital abnormalities.

This study underscores the need for clinicians to maintain a high index of suspicion for HNF1B-associated nephropathy in patients with complex kidney and systemic findings, particularly when there is a family history of similar conditions. It advocates for early genetic evaluation to bridge diagnostic delays, improve patient outcomes, and enable family-wide screening. By illustrating, this work emphasizes the critical role of integrating clinical, radiological, and genetic insights in rare diseases.

## Data Availability

The original contributions presented in the study are included in the article/Supplementary material, further inquiries can be directed to the corresponding author.

## References

[1] Horikawa Y Iwasaki N Hara M Furuta H Hinokio Y Cockburn BN . Mutation in hepatocyte nuclear factor-1 beta gene (HNF1B) associated with MODY. Nat Genet. (1997) 17:384–5. doi: 10.1038/ng1297-384 9398836

[2] Verhave JC Bech AP Wetzels JF Nijenhuis T. Hepatocyte nuclear factor 1beta-associated kidney disease: more than renal cysts and diabetes. J Am Soc Nephrol. (2016) 27:345–53. doi: 10.1681/ASN.2015050544 26319241 PMC4731131

[3] Ferre S Bongers EM Sonneveld R Cornelissen EA van der Vlag J van Boekel GA . Early development of hyperparathyroidism due to loss of PTH transcriptional repression in patients with HNF1beta mutations? J Clin Endocrinol Metab. (2013) 98:4089–96. doi: 10.1210/jc.2012-3453 23979948

[4] Clissold RL Hamilton AJ Hattersley AT Ellard S Bingham C. HNF1B-associated renal and extra-renal disease—an expanding clinical spectrum. Nat Rev Nephrol. (2015) 11:102–12. doi: 10.1038/nrneph.2014.232 25536396

[5] Kolatsi-Joannou M Bingham C Ellard S Bulman MP Allen LIS Hattersley AT . Hepatocyte nuclear factor-1β: a new kindred with renal cysts and diabetes and gene expression in normal human development. J Am Soc Nephrol. (2001) 12:2175–80. doi: 10.1681/ASN.V12102175 11562418

[6] Faguer S Decramer S Chassaing N Bellanné-Chantelot C Calvas P Beaufils S . Diagnosis, management, and prognosis of HNF1B nephropathy in adulthood. Kidney Int. (2011) 80:768–76. doi: 10.1038/ki.2011.225 21775974

[7] Raaijmakers A Corveleyn A Devriendt K van Tienoven TP Allegaert K Van Dyck M . Criteria for HNF1B analysis in patients with congenital abnormalities of kidney and urinary tract. Nephrol Dial Transplant. (2015) 30:835–42. doi: 10.1093/ndt/gfu370 25500806

[8] Bingham C Hattersley AT. Renal cysts and diabetes syndrome resulting from mutations in hepatocyte nuclear factor-1beta. Nephrol Dial Transplant. (2004) 19:2703–8. doi: 10.1093/ndt/gfh348 15496559

[9] Dubois-Laforgue D Cornu E Saint-Martin C Coste J Bellanné-Chantelot C Timsit J . Diabetes, associated clinical spectrum, long-term prognosis, and genotype/phenotype correlations in 201 adult patients with hepatocyte nuclear factor 1B (HNF1B) molecular defects. Diabetes Care. (2017) 40:1436–43. doi: 10.2337/dc16-2462 28420700

[10] Lopes AM Teixeira S. New-onset diabetes after kidney transplantation revealing HNF1B-associated disease. Endocrinol Diabetes Metab Case Rep. (2021) 2021:20–0165. doi: 10.1530/EDM-20-0165 33522494 PMC7849473

[11] Edghill EL Bingham C Slingerland AS Minton JAL Noordam C Ellard S . Hepatocyte nuclear factor-1 beta mutations cause neonatal diabetes and intrauterine growth retardation: support for a critical role of HNF-1beta in human pancreatic development. Diabet Med. (2006) 23:1301–6. doi: 10.1111/j.1464-5491.2006.01999.x 17116179

[12] Haldorsen IS Vesterhus M Raeder H Jensen DK Søvik O Molven A . Lack of pancreatic body and tail in HNF1B mutation carriers. Diabet Med. (2008) 25:782–7. doi: 10.1111/j.1464-5491.2008.02460.x 18644064

[13] Van der Made CI Hoorn EJ de la Faille R Karaaslan H Knoers NV Hoenderop JG. Hypomagnesemia as first clinical manifestation of ADTKD-HNF1B: a case series and literature review. Am J Nephrol. (2015) 42:85–90. doi: 10.1159/000439286 26340261

[14] Chandra S Srinivasan S Batra J. Hepatocyte nuclear factor 1 beta: a perspective in cancer. Cancer Med. (2021) 10:1791–804. doi: 10.1002/cam4.3676 33580750 PMC7940219

[15] Bingham C Ellard S 't Hoff WG Simmonds HA Marinaki AM Badman MK . Atypical familial juvenile hyperuricemic nephropathy associated with a hepatocyte nuclear factor-1beta gene mutation. Kidney Int. (2003) 63:1645–51. doi: 10.1046/j.1523-1755.2003.00903.x 12675839

[16] Gambella A Kalantari S Cadamuro M Quaglia M Delvecchio M Fabris L . The landscape of HNF1B deficiency: a syndrome not yet fully explored. Cells. (2023) 12:307. doi: 10.3390/cells12020307 36672242 PMC9856658

[17] Kotalova R Dusatkova P Cinek O Dusatkova L Dedic T Seeman T . Hepatic phenotypes of HNF1B gene mutations: a case of neonatal cholestasis requiring portoenterostomy and literature review. World J Gastroenterol. (2015) 21:2550–7. doi: 10.3748/wjg.v21.i8.2550 25741167 PMC4342936

[18] Faguer S Esposito L Casemayou A Pirson Y Decramer S Cartery C . Calcineurin inhibitors downregulate HNF-1beta and may affect the outcome of HNF1B patients after renal transplantation. Transplantation. (2016) 100:1970–8. doi: 10.1097/TP.0000000000000993 26555949

[19] Aboudehen K Noureddine L Cobo-Stark P Avdulov S Farahani S Gearhart MD . Hepatocyte nuclear factor-1beta regulates urinary concentration and response to hypertonicity. J Am Soc Nephrol. (2017) 28:2887–900. doi: 10.1681/ASN.2016101095 28507058 PMC5619957

[20] Bockenhauer D Jaureguiberry G. HNF1B-associated clinical phenotypes: the kidney and beyond. Pediatr Nephrol. (2016) 31:707–14. doi: 10.1007/s00467-015-3142-2 26160100

