# Supplementary figures and images for: Case Report: Nephrocalcinosis from pancreatic hypoplasia in HNF1B disease: a multigenerational expression with genetic confirmation in the youngest generation
Source: Front Med (Lausanne). 2026 Jan 30;12:1671893. doi: 10.3389/fmed.2025.1671893 (PMC12903773; doi:10.3389/fmed.2025.1671893)

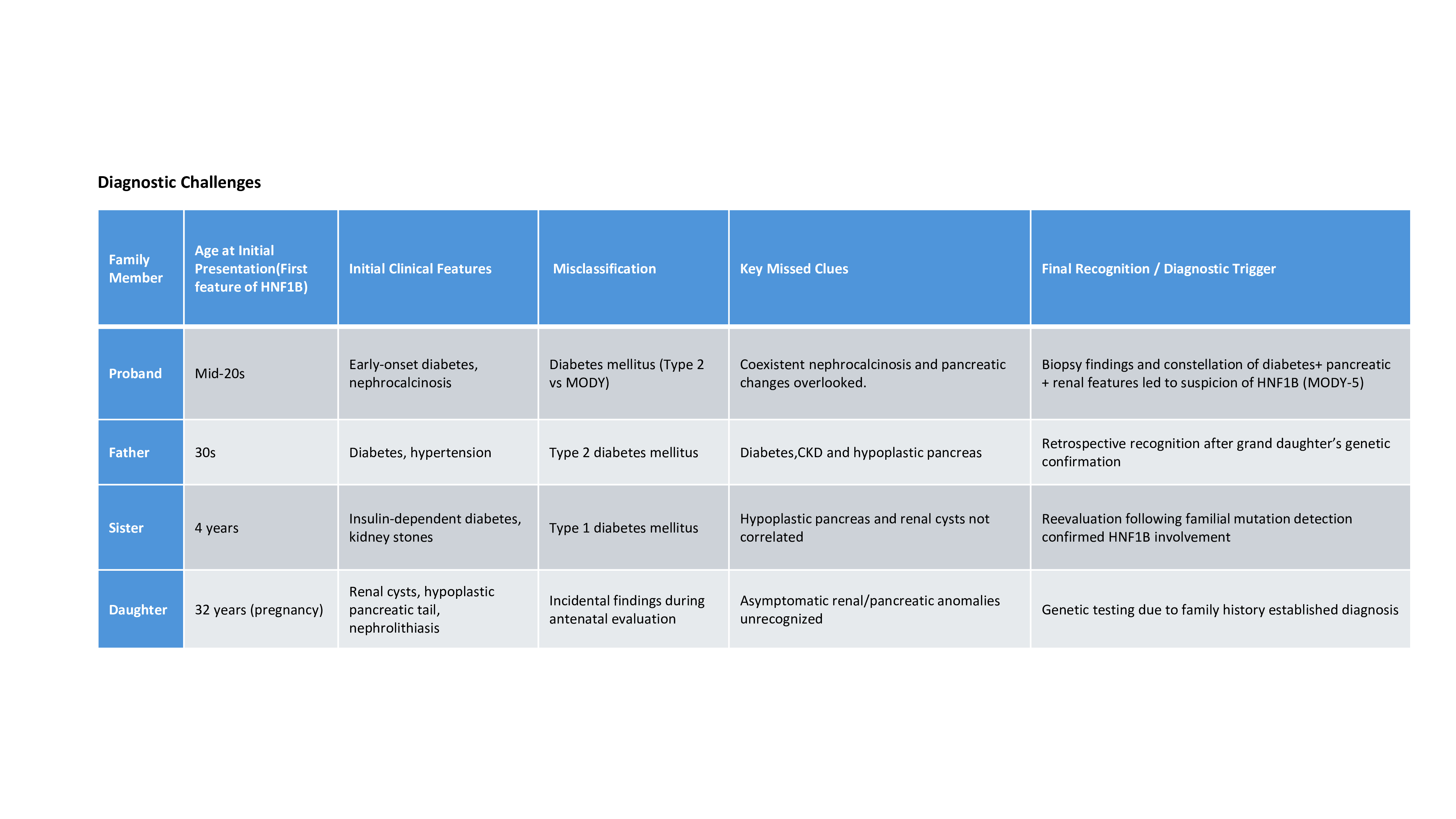

Supplement: Supplementary file 1 [file Image_1.jpeg]
